# Effects of melatonin and metformin in preventing lysosome-induced autophagy and oxidative stress in rat models of carcinogenesis and the impact of high-fat diet

**DOI:** 10.1038/s41598-022-08778-w

**Published:** 2022-03-23

**Authors:** Natalia Kurhaluk, Halyna Tkachenko

**Affiliations:** Department of Biology, Institute of Biology and Earth Sciences, Pomeranian University in Słupsk, Arciszewski Str., 22b, 76-200 Słupsk, Poland

**Keywords:** Biochemistry, Cancer, Cell biology, Chemical biology, Physiology

## Abstract

Imbalanced glucose tolerance and insulin resistance remain important as high cancer risk factors. Metformin administration to diabetic patients may be associated with a reduced risk of malignancy. The combined effects of the hormone melatonin and metformin in oncology practice have shown positive results. The relevance of our study is to find out the role of specific biomarkers of lysosome destruction and oxidative stress data in carcinogenesis models. The present study was designed to investigate the comparative synergic effect of peroral antidiabetic metformin (MF) and pineal hormone melatonin (MEL) administered alone and in combination in two different rat’s models of mammary tumour proliferation in vivo (*N*-methyl-*N*-nitrosourea, NMU or 7,12-dimethylbenz[a]anthracene, DMBA). We have studied the processes of lysosomal destruction (alanyl aminopeptidase AAP, leucyl aminopeptidase LAP, acid phosphatase AcP, β-*N*-acetylglucosaminidase NAG, β-galactosidase β-GD and β-glucuronidase β-GR) caused by evaluated oxidative stress in three types of tissues (liver, heart, and spleen) in female Sprague–Dawley rats fed a high-fat diet (10% of total fat: 2.5% from lard and 7.5% from palm olein). Our results revealed an increase in the activity of the studied lysosomal enzymes and their expression in a tissue-specific manner depending on the type of chemical agent (NMU or DMBA). MANOVA tests in our study confirmed the influence of the three main factors, type of tissue, chemical impact, and chemopreventive agents, and the combinations of these factors on the lysosomal activity induced during the process of cancerogenesis. The development and induction of the carcinogenesis process in the different rat models with the high-fat diet impact were also accompanied by initiation of free-radical oxidation processes, which we studied at the initial (estimated by the level of diene conjugates) and final (TBARS products) stages of this process. The combined effects of MEL and MF for the two models of carcinogenesis at high-fat diet impact for AAP, LAP, and AcP showed a significant synergistic effect when they impact together when compared with the effects of one substance alone (either MEL or MF) in the breast cancer model experiments. Synergistic effects of limiting destructive processes of lysosomal functioning β-GD enzyme activity we obtained in experiments with MEL and MF chemoprevention for both models of carcinogenesis for three tissues. The statistical SS test allowed us to draw the following conclusions on the role of each lysosomal parameter analyzed as an integral model: NAG > AcP > β-GD > β-GR > AAP > LAP.

## Introduction

Cancer is the second leading cause of death worldwide (after cardiovascular disease). Cancer is responsible for 25% of all deaths^1^. Breast cancer is the most common female malignancy in Europe (464,000 cases, 13.5% of all cancer cases)^2^, with over 10,000 new cases each year, especially in young women^3^. Scientific studies have reported that cancer patients often have symptoms of metabolic syndrome, a component of which is glucose intolerance and diabetes mellitus type 2. Therefore, evaluation of oral anti-diabetic preparations with anti-tumor properties is particularly important. It is known that the risk of malignant transformation may be increased by various other lifestyle-related factors, e.g. working at night, insomnia, poor diet, and high dietary fat content^4^. Hence, elucidation of the potential mechanisms responsible for the increased production of reactive oxygen species in cancer cells in highly developed countries is an important component of basic research.

In recent decades, a number of promising studies have been focused on various drug compounds that actively influence hormonal and metabolic changes in the organism in order to reduce the risk of malignant neoplasms^5^. Although there are potential benefits in using chemoprevention to prevent cancer, there has been little interest in the scientific community. Many compounds have been proposed as potential chemopreventative agents, but only a few have been adopted for routine clinical use. Equally important is the attempt to increase the effectiveness of the treatment of cancer patients through elimination of metabolic syndrome and related endocrine disorders. It is known that many cancer patients develop insulin resistance, impaired glucose tolerance, activation of the insulin-like growth factor system, and visceral obesity, which may be associated with the global problem of excessive carbohydrate consumption and the so-called obesity epidemic. The research interest in metformin (MF) has increased in recent years due to the encouraging results of in vitro and in vivo animal experiments demonstrating the pronounced anti-tumor activity of MF^6,7^. However, the mechanism of its antitumor action is still unclear. It is assumed that, by activating adenosine monophosphate-dependent protein kinase (a key enzyme in the regulation of cellular energy supply), MF indirectly inhibits the activity of mTOR (a central regulator of protein synthesis and cell growth). The action of MF is associated with a decrease in glucose levels and an increase in glucose consumption by tumor cells resulting in a decrease in insulin levels and significantly affecting the insulin-like growth factor system, which is another potential target^8^.

Melatonin (MEL), i.e. a pineal hormone acting as a chronobiotic and reproduction regulator, is one of the oldest signal molecules. MEL is a potent antioxidant^9–11^ and exerts anti-inflammatory^12^, immunomodulative, antiproliferative, proapoptotic, and antiangiogenic effects^13^. In addition to the pineal gland, melatonin is synthesized in immune cells. MEL properties are involved in its oncostatic effect, which was first reported in the MCF-7 mammary adenocarcinoma line; later on, its preventive and therapeutic effect was reported in many experimental neoplasia models and human cancers^14,15^. Induced by lifestyle-related factors, especially women's night work and exposure to such xenobiotics as alcohol^4,16^, significant reductions in synthesis of MEL, i.e. a potential antioxidant and anti-cancer agent, have become widely underlined in cancer pathogenesis. Researchers have found preventive effects of MEL administered alone and in combination with other chemopreventives in female rat mammary carcinogenesis^17^. The results indicate that the preventive-curative treatment (i.e. MEL administration before and after carcinogenesis induction) is the most suitable approach, as proved by the increased latency and survival time, and a combination with other oncostatic substances is preferable.

Studies have established that reactive oxygen species (ROS) are involved in the etiology and progression of breast cancer. Biomarkers of oxidative stress such as malondialdehyde, 8-isoprostane, carbonyl oxidation products of proteins such as diene conjugates and DNA adducts have been shown to be frequently detected in breast cancer patients^18^. High levels of ROS in cancer cells during anticancer therapy can lead to a variety of biological responses such as cell adaptation, increased proliferation rates, DNA mutation formation, genetic instability and resistance to these drugs^19^. Thus, the assessment and investigation of the stages of oxidative stress in cancer cells can both contribute to tumour development and may be useful in the search for new therapeutic strategies to treat cancer^20^. A number of studies have discussed the dysregulation of ROS metabolism in breast cancer patients as detected by various biomarkers in plasma or various blood cells, including red blood cells and platelets^21^.

Lysosomes are multifunctional cellular organelles that not only provide degradation of macromolecules by lumenal acidic hydrolases but also participate in the regulation of cellular metabolism, maintenance of ion homeostasis, and induction of programmed cell death^22^. Of particular interest are studies of the role of this compartment in the development of cancerous diseases with varied genesis. A number of studies have shown changes induced by free radical processes, relationships between oxidative stress and lysosomal destruction^23^, changes in lysosomes occurring in the process of malignant cell transformation, and the role of this compartment in such a key stage of oncogenesis as metastasis mediated by degradation of the extracellular matrix^24^. Metabolic and morphological cellular changes during oncogenesis lead to pH-dependent redistribution of lysosomes within the cell accompanied by secretion of lysosomal proteases (cathepsins) into the extracellular space. The study of cancer pathogenesis confirms that lysosomes are dynamic compartments changing their localization, functional orientation, and composition depending on the conditions of the cellular microenvironment^25^. At the same time, the transformations are associated with both metabolic (transition to the glycolytic type of energy metabolism) and morphological (formation of invadopodium) changes occurring in cells during oncogenesis. As the sources of cysteine, serine, and arginine cathepsins are directly involved in the degradation of extracellular matrix proteins or triggering proteolytic cascades through the activation of other proteases, lysosomes mediate cancer cell growth progression and metastasis^26^. Therefore, clarification of the mechanisms of cancer transformation and the role of lysosomal destruction biomarkers in the induction of oxidative stress is a relevant topic. The significance of our study is to elucidate the role of specific biomarkers of lysosomal destruction and oxidative stress data in different models of carcinogenesis.

We decided to extend and complement the knowledge of the synergistic MEL and MF effects observed in our previous studies and by other authors in cancer models^11,12,17^. We performed a comparative analysis of the effects of these drugs in two different cancer models (induced by *N*-methyl-*N*-nitrosourea, NMU and 7,12-dimethylbenz[a]anthracene, DMBA) on lysosomal enzyme status in relation to the induction of oxidative stress in different types of tissues. Tissues differ in their sensitivity to oncostatic agents due to the different levels of metabolic processes, hematopoiesis intensity, and morphological characteristics of cells. Our aim was to analyze the effect of MF administered alone and in combination with MEL in chemically induced mammary carcinogenesis in female Sprague–Dawley rats fed a high-fat diet.

## Materials and methods

### Animals and experimental design

The experiments were conducted with the Guidelines of the European Union Council, including Directive 2010/63/EU, the current laws in Slovakia and Poland, experiments were performed in accordance with relevant institutional and national guidelines and regulations for the care and use of laboratory animals. The study was conducted according to the guidelines of the declaration of Helsinki and approved by the State Veterinary and Food Administration of the Slovak Republic (accreditation No. Ro-2054/13-221). The animals were treated and sacrificed in a humane manner according to the principles provided in the Law No. 289/2003, 489/2003, and 23/2009 of Slovak Republic for the Care and Use of Laboratory Animals. Animals were treated in accordance with the principles established in the Law No. 377/2012 and 436/2012 of Slovak Republic for the Care and Use of Laboratory Animals. The experiment was approved by the State Veterinary and Food Administration of the Slovak Republic (accreditation No. Ro. 2054/13-221, 2368/12-221 and 2765/11-221/3).

Female rats of the sensitive Sprague–Dawley strain (AnLab, Prague, Czech Republic) aged 30 days were used in the experiment. This strain is most commonly used in in vivo mammary carcinogenesis models, and other strains (e.g. Wistar) are little sensitive to mammary tumor induction by chemocarcinogens. The animals were adapted to standard vivarium conditions with a temperature of 23 ± 2 °C, 60–70% relative humidity, and an artificial light–dark cycle of 12: 12 h (lights on from 7 a.m., light intensity 150 lx/cage). During the experiment, the animals (6/cage) were fed a high-fat diet (10% of total fat: 2.5% from lard, 7.5% from palm olein; Biofer, Slovak Republic) and tap water or a MEL solution ad libitum. We have previously described this type of diet in the study^4^. Mammary carcinogenesis was induced in two intraperitoneal doses of *N*-methyl-*N*-nitrosourea (each per 50 mg/kg, with one-week interval between the doses) or in a single intragastric dose of 7,12-dimethylbenz[a]anthracene (DMBA, 20 mg/rat) administered in a sensitive period of mammary gland development (postnatal days 40–50)^27^. Mammary carcinogenesis was induced by *N*-methyl-*N*-nitrosourea (NMU, cat. no. N4766; Sigma-Aldrich, Deisenhofen, Germany) administered intraperitoneally (50 mg/kg) body weight) on postnatal day 42. The NMU solution was freshly prepared before administration by dissolving NMU in 0.9% NaCl (the average volume dose per rat was 0.5 ml).

The chemopreventive substances ( MF and MEL) were administered in a long time (12 days before and 16–18 weeks after carcinogenesis induction, until experiment termination) in order to cover the initiation and promotion/progression of the carcinogenesis stage. MF was administered in the diet at a concentration of 2000 ppm (cat. no. M5250, Sigma-Aldrich, Deisenhofen, Germany) in tap water at a concentration of 20 mg/L daily from 15:00. to 8:00 (only water from 8:00 to 15:00). The choice of the doses of the chemopreventives was based on our previous experiments^17,27^.

MEL was administered every day in drinking water between 15:00 and 8:00 (between 8:00 and 15:00, the animals received tap water only). We tried to simulate the natural MEL rhythm by intermittent administration (with a peak concentration during the dark phase of the day). The animals were fed the high-fat diet with 10% of fat (with predominance of saturated fatty acids) in order to simulate the lifestyle in developed countries (western-type diet), as described earlier^28^. The effect of the combination (MF and MEL) was analyzed in animals fed a standard diet as well (in the NMU and DMBA model) as a follow-up to our previous experiments, where these substances were administered alone.

The animals were assigned randomly to nine experimental groups: 1) Control group (n = 10), 2) NMU model (n = 18), 3) DMBA model (n = 18), 4) MF + NMU (n = 18), 5) MF + DMBA (n = 18), 6) Mel + NMU (n = 18), 7) MEL + DMBA (n = 18), 8) MF + MEL + NMU, chemoprevention with a combination of metformin and melatonin (n = 18), 9) MF + MEL + DMBA, chemoprevention with a combination of metformin and melatonin (n = 18).

The animals were weighed weekly and palpated to register the presence, number, location, and size of each palpable tumor. Food and water intake over the 24-h period was monitored within the 4th, 9th, and 14th week of the experiment (dated from NMU and DMBA administration). In the last 16th week of the experiment, the animals were killed by quick decapitation and mammary tumors, selected organs, and tissues were removed for further evaluation. Tissue samples were stored in liquid nitrogen medium and transported to Pomeranian University in Słupsk, where the biomarkers of lysosomal functioning and oxidative stress were analyzed.

The design of the NMU and DMBA model, MF and MEL administration in these conditions, and animal body weight gain, food and water intake, and basic tumor growth parameters (incidence, latency, frequency per group and animal, and average and cumulative tumor volume) were evaluated by a research team of well-known experts from Pavol Jozef Šafárik University in Košice in international collaboration. The principal investigator was Bianka Bojkova, Ph.D., Department of Animal Physiology, Institute of Biology and Ecology, Faculty of Science, Pavol Jozef Šafárik University in Košice, Košice, Slovak Republic.

### Tissue homogenate preparation

The tissue specimens from liver, heart, and spleen were weighed, washed in ice-cold buffer, and minced. The minced tissue was rinsed with cold isolation buffer to remove blood and homogenized in a glass Potter–Elvehjem homogenizer with a motor-driven Teflon pestle on ice. The isolation buffer consisted of 120 mM KCl, 2 mM K_2_CO_3_, 10 mM HEPES, and 1 mM EDTA; pH was adjusted to the value of 7.2 with KOH. Tissue homogenates were used for determination of the content of conjugated dienes (A_233_, nmol/mg protein) and TBARS products (nmol/mg protein), as well as the activity of such antioxidant enzymes as superoxide dismutase SOD, catalase CAT, glutathione reductase (GR), and glutathione peroxidase GPx in the liver, heart, spleen. The methods used for tissue homogenate preparations have been described by us previously^11^. The Bradford method with bovine serum albumin as a standard was used for quantification of proteins. Absorbance was recorded at 595 nm.

### Tissue isolation for lysosomal enzyme assays

The selected organs (liver, heart, spleen) were removed from the rats after decapitation. The tissue samples were excised, weighted, washed in ice-cold buffer, and minced. The minced tissues were rinsed with cold isolation buffer 0.15 M KCL to remove blood and homogenized in a glass Potter–Elvehjem homogenizer with a motor-driven Teflon pestle on ice. The isolation buffer consisted of 0.25 M sucrose and 2 mM EDTA; the pH was adjusted to 7.0 with KOH. The homogenates of several livers, hearts, and spleens 20% (w/v) were prepared to next differential centrifugation according to the method described by DeMartino and Goldberg^29^. The supernatant fractions after centrifugation were saved and used after resuspension in 50 mM acetic acid/sodium acetate buffer at pH 5.0. These isolated fractions of each tissue type were homogenized and subjected to two freeze–thaw cycles.

### Biochemical assays

#### Thiobarbituric acid reactive substances (TBARS) assay

TBARS were measured with the method proposed by Kamyshnikov^30^ using the maximal absorbance at 540 nm. The TBARS level was expressed in nmol of malondialdehyde (MDA) per nmol of MDA per mg protein.

#### Superoxide dismutase activity assay

Superoxide dismutase (SOD) activity in the supernatant was determined according to Kostiuk et al.^31^. SOD activity was assessed by its ability to dismutate the superoxide produced during quercetin auto-oxidation in an alkaline medium (pH 10.0). Absorbance at 406 nm was measured immediately and after 20 min. The activity was expressed in units of SOD per mg of protein.

#### Catalase activity assay

Catalase (CAT) activity was determined by measuring the decrease in H_2_O_2_ in the reaction mixture with the method developed by Koroliuk et al.^32^. One unit of CAT activity was defined as the amount of enzyme required for decomposition of 1 μmol H_2_O_2_ per min per mg of protein.

#### Glutathione reductase activity assay

Glutathione reductase (GR) activity in the blood and tissues was measured according to the method proposed by Glatzle et al.^33^. The enzymatic activity was assayed spectrophotometrically by measuring NADPH consumption. A blank without NADPH was used and the GR activity was expressed as nmol NADPH per min per mg of protein.

#### Glutathione peroxidase activity assay

Glutathione peroxidase (GPx) activity was determined by detection of nonenzymatic utilization of reduced glutathione (GSH) as the reacting substrate at 412 nm after incubation with 5,5-dithiobis-2-nitrobenzoic acid (DTNB) according to the method described by Moin^34^. The GPx activity was expressed as nmol GSH per min per mg of protein.

#### Lysosomal enzyme assays

The activity of alanyl aminopeptidase and leucyl aminopeptidase was determined spectrophotometrically as Fast Blue BB salt (4-benzoyloamino-2,5-diethoxybenzene-diazonium chloride) derivatives at 540 nm according to method proposed by McDonald and Barrett^35^. The reaction was initiated using 50 μl of the sample and 500 μl of the substrate incubation media with DMF (Serva, Germany). After 60-min incubation at 37 °C, pH 6.0, 500 μl of stop buffer consisting of Fast Blue BB Salt dissolved in 2% Tween 20 were added (Sigma, USA). Absorbance was measured at 540 nm. L-alanyl-2-naphthylamine in 0.1 M PBS buffer was used as a substrate for determination of alanyl aminopeptidase activity. L-leucyl-2-naphthylamine in 0.1 M PBS pH 7.0 buffer was used as a substrate for determination of leucyl aminopeptidase activity.

The activities of other lysosomal enzymes, e.g. acid phosphatase and β-*N*-acetylglucosaminidase, were determined spectrophotometrically as 4-nitrophenyl derivatives at 420 nm as described by Barrett and Heath^36^. The activities of enzymes were expressed in nmol/h and in mg protein assessed with the Bradford method (Bradford, 1976) with bovine serum albumin as a standard. Absorbance of proteins was recorded at 595 nm.

### Statistical analysis

The results are expressed as means ± S.D. All variables were tested for normal distribution using the Kolmogorov–Smirnov and Lilliefors tests (*P* > 0.05), and homogeneity of variance was checked using Levene’s test. The significance of differences in the level of lysosomal enzymes, conjugated dienes, 2-thiobarbituric acid reactive substances, and antioxidant enzyme activity between the control and all investigated groups were examined using Student’s t-test. Differences were considered significant at *P* < 0.05. In addition, the associations between the data were evaluated using Pearson's correlation analysis. MANOVA tests were used in our study to analyze the influence of the three main factors: the type of tissue, chemical impact, and chemopreventive agents and the combinations of these factors on the lysosomal activity and biomarkers of oxidative stress induced during the carcinogenesis process. All statistical calculations were performed on separate data from each animal with Statistica 13.3.0 software (TIBCO Software Inc.).

## Results

### Lysosomal enzymes

The alanyl aminopeptidase (AAP) enzyme has been associated with the growth of various human and animal cancers and is indicated as a marker and target of anti-cancer research. Its expression is dysregulated in inflammatory diseases and cancers (solid and hematologic tumors), as convincingly demonstrated in many studies^37,38^. Therefore, we began our research by studying the activity of this enzyme. AAP is a Zn^2+^-dependent membrane-bound ectopeptidase preferentially degrading proteins and peptides with an N-terminal neutral amino acid. The design of the two independent cancer models (NMU and DMBA) in Sprague–Dawley strain rats fed the high-fat diet allowed us to determine that the activity of AAP and the therapeutic agents did not produce statistically significant changes in the liver tissue. The results of this experimental series are shown in Table 1.

**Table 1:** Effect of melatonin and metformin on alanyl- and leucyl aminopeptidase activity (nmol h^−1^ mg^−1^ protein) in the liver, heart, and spleen in female Sprague-Dawley rats subjected to a high-fat diet in the mammary carcinogenesis model induced by *N*-methyl-*N*-nitrosourea (NMU) or dimethylbenzanthracene (DMBA). Metformin (MF) or melatonin (MEL) was administered alone and in combination (MF + MEL).

Parameters/ Groups	Liver	Heart	Spleen	Liver	Heart	Spleen
	AAP, nmol h^−1^ mg^−1^ protein			LAP, nmol h^−1^ mg^−1^ protein		
Control	799.88 ± 167.48	166.69 ± 25.9	121.82 ± 8.63	55.17 ± 7.70	221.79 ± 20.11	241.69 ± 20.14
NMU	976.10 ± 135.39	178.3 ± 21.25	158.44 ± 19.31^a^	152.21 ± 25.41^a^	236.33 ± 38.95	264.17 ± 31.12
DMBA	1078.33 ± 218.20	241.14 ± 20.14^aa^	148.54 ± 16.18^aa^	146.20 ± 32.01^aa^	258.01 ± 28.21	274.95 ± 31.12
MF + NMU	1016.80 ± 190.60	212.46 ± 45.14	155.20 ± 26.18	116.85 ± 7.89^b^	237.92 ± 20.47	255.92 ± 29.49
MF + DMBA	845.56 ± 198.12	201.52 ± 25.4	138.95 ± 22.41	101.25 ± 11.20	244.50 ± 24.69	258.72 ± 19.49
MEL + NMU	968.19 ± 144.58	285.11 ± 68.31^c^	174.31 ± 26.29	118.25 ± 9.58^c^	232.16 ± 18.72	232.16 ± 21.10
MEL + DMBA	985.22 ± 214.02	265.21 ± 33.01^cc^	154.22 ± 47.9	98.47 ± 9.52^cc^	219.33 ± 23.01	255.92 ± 39.49
MF + MEL + NMU	946.50 ± 134.96	213.43 ± 35.42^e^	121.82 ± 17.96^e^	121.11 ± 10.21	242.11 ± 33.01	232.01 ± 17.05
MF + MEL + DMBA	857.23 ± 158.12	190.47 ± 24.11^ee^	132.15 ± 17.91	78.04 ± 8.21^dd^	199.07 ± 17.58	199.66 ± 18.47^dd^

Note. Data are expressed as means ± S.D.

Significant differences *P* < 0.05 are designated as follows: a—NMU group vs. control group; aa—DMBA group vs. control group; b—MF + NMU vs. NMU group; bb—MF + DMBA vs. DMBA group; c—MEL + NMU vs. NMU group; cc—MEL + DMBA vs. DMBA group; d—MEL + MF + NMU vs. MF + NMU group; dd—MEL + MF + DMBA vs. MF + DMBA group; e—MEL + MF + NMU vs. MEL + NMU group; ee—MEL + MF + DMBA vs. MEL + DMBA group.

AAP, alanyl aminopeptidase; LAP, leucyl aminopeptidase.

NMU, *N*-methyl-*N*-nitrosourea; DMBA, dimethylbenzanthracene; MF, metformin; MEL, melatonin.

A comparative analysis of the effects of carcinogenesis induction in the cardiac tissue showed a statistically significant increase in AAP activity in the DMBA model compared to the control animal group (Table 1). In the case of melatonin effects in both models of carcinogenesis in the cardiac tissue, a statistically significant increase in AAP activity was found, which was more pronounced in the series of MEL + NMU experiments compared to the carcinogenesis model group. The combined effects of MEL and MF in the two models of carcinogenesis with the high-fat diet impact on AAP showed a significant synergistic effect of the combination treatment, compared with the effects of one substance alone (either MEL or MF) in the breast cancer model experiments. The membrane processes of chemical carcinogenesis induction assessed by an increase in AAP activity in splenic tissue caused by DMBA showed high sensitivity of this tissue differing statistically significantly from that in the NMU treatment. A synergistic effect of MEL and MF on the model of NMU carcinogenesis was observed only in this case. The value of AAP activity of the enzyme was similar to the control values (Table 1).

Membrane processes of chemical induction of carcinogenesis with the high-fat diet impact assessed by the activity of another lysosomal enzyme, i.e. LAP, were shown by us in a series of studies (Table 1). Leucine aminopeptidase is involved in the progression and metastasis of several cancers, as shown in several studies^39,40^. We observed a marked (three-fold) increase in LAP enzyme activity in the hepatic tissue without impairment of this enzyme in the heart and spleen lysosomes. The activation of membrane-dependent processes of lysosomal LAP functioning in the hepatic tissue was reduced statistically significantly in the series of experiments using MF and MEL in the NMU model and MEL in the DMBA model. A marked synergistic effect of both drugs was noted in the DMBA model of carcinogenesis with the high-fat diet impact, where a statistical reduction of LAP activity approaching the level of the control group of animals was observed. It should be noted that a similar synergistic effect was observed in the splenic tissue, compared to the MF + DMBA group of animals (Table 1).

The convincing link between chronic inflammation, breast cancer risk, and acid phosphatase activity shown by other authors^41^ was the basis for our next step in elucidating the role of the acid phosphatase (AcP) enzyme in a comparative analysis of tissue exposure and carcinogenesis models in animals. The results of this experimental series are shown in Table 2. We identified tissue-dependent effects on AcP in lysosomes in the chemical model of NMU and DMBA carcinogenesis with the high-fat diet impact. Although we showed a statistically significant two-fold increase in AcP activity in the hepatic and splenic tissues, we did not observe any changes in the activity of this enzyme in the heart. In the case of the AcP activity in the DMBA model under the influence of MEL and MF, we obtained statistically significant limiting effects on lysosome destruction associated with an increase in the activity of this enzyme in the NMU and DMBA carcinogenesis variants. The synergistic effects of MEL and MF chemoprevention in these carcinogenesis models with the high-fat diet impact were associated with a decrease in AcP activity caused by both NMU and DNBA exposure alone, as observed for lysosomes in both hepatic and splenic tissues (Table 2).

**Table 2:** Effect of melatonin and metformin on acid phosphatase and β-*N*-acetylglucosaminidase activity (nM/h/mg protein) in the liver, heart and spleen in female Sprague-Dawley rats subjected to a high-fat diet in the mammary carcinogenesis model induced by *N*-methyl-*N*-nitrosourea (NMU) or dimethylbenzanthracene (DMBA). Metformin (MF) or melatonin (MEL) was administered alone and in combination (MF + MEL).

Parameters/ Groups	Liver	Heart	Spleen	Liver	Heart	Spleen
	AcP, nmol h^−1^ mg^−1^ protein			NAG, nmol h^−1^ mg^−1^ protein		
Control	572.65 ± 67.49	603.00 ± 49.86	280.83 ± 37.98	255.23 ± 37.73	582.13 ± 40.34	205.05 ± 2.14
NMU	1036.33 ± 105.21^a^	596.58 ± 68.31	458.04 ± 39.31^a^	770.63 ± 55.41^a^	693.28 ± 51.14^a^	310.25 ± 11.92^a^
DMBA	1241.78 ± 109.16^aa^	641.18 ± 68.31	378.54 ± 46.18^aa^	684.03 ± 52.01^aa^	593.12 ± 69.62	245.95 ± 23.20^aa^
MF + NMU	916.52 ± 128.41	740.42 ± 65.14	425.22 ± 56.18	530.11 ± 47.89^b^	687.16 ± 42.47	283.94 ± 23.82
MF + DMBA	878.56 ± 98.12^bb^	630.52 ± 85.40	338.04 ± 42.41	608.25 ± 11.20	736.14 ± 54.69^bb^	208.42 ± 19.47
MEL + NMU	903.19 ± 84.57	721.23 ± 58.41	338.31 ± 46.29	682.25 ± 59.48	778.91 ± 58.72	255.78 ± 30.10
MEL + DMBA	985.21 ± 94.02^cc^	665.24 ± 83.08	424.22 ± 57.9	498.47 ± 19.52^cc^	719.33 ± 43.01	187.16 ± 10.49^cc^
MF + MEL + NMU	711.50 ± 84.96^d^	643.48 ± 35.42	229.82 ± 19.92^d,e^	444.32 ± 40.21^d,e^	504.26 ± 43.01^d,e^	139.58 ± 17.05^d,e^
MF + MEL + DMBA	749.23 ± 58.18^dd,ee^	590.41 ± 74.11	242.18 ± 34.91^dd,ee^	382.14 ± 48.21^dd,ee^	521.45 ± 57.58^dd,ee^	147.21 ± 18.07^dd^

Note. Data are expressed as means ± S.D.

Significant differences *P* < 0.05 are designated as follows: a—NMU group vs. control group; aa—DMBA group vs. control group; b—MF + NMU vs. NMU group; bb—MF + DMBA vs. DMBA group; c—MEL + NMU vs. NMU group; cc—MEL + DMBA vs. DMBA group; d—MEL + MF + NMU vs. MF + NMU group; dd—MEL + MF + DMBA vs. MF + DMBA group; e—MEL + MF + NMU vs. MEL + NMU group; ee—MEL + MF + DMBA vs. MEL + DMBA group.

AcP, acid phosphatase; NAG, β-*N*-acetylglucosaminidase.

NMU, *N*-methyl-*N*-nitrosourea; DMBA, dimethylbenzanthracene; MF, metformin; MEL, melatonin.

Ramessur et al.^42^ presented convincing arguments for the link between successful cancer cell invasion and the role of β-*N*-acetylglucosaminidase in breast cancer models, which determined the further steps in our investigations. The activity of β-*N*-acetylglucosaminidase (NAG) in the lysosomes in the three tissues is shown in Table 2. In this series of studies of the analyzed models of carcinogenesis with the high-fat diet impact, we demonstrated a two- and even three-fold increase in the activity of the NAG enzyme, which may indicate a high level of destructive processes caused by the cancer induction in the rats. In the hepatic tissue, MF used in the NMU model and MEL in the DMBA model caused normalization of lysosome functioning processes related to the NAG enzyme, compared to the data from the NMU or DMBA models of carcinogenesis. We also obtained similar relationships in the case of the splenic tissue in the MEL chemoprevention experiments and the DMBA model with the high-fat diet impact. However, the effects of MF in the DMBA model of carcinogenesis induction were associated with a statistically significant increase in NAG enzyme activity, which was reversible, i.e. it was significantly decreased when the two drugs were already active. Thus, we obtained a synergistic positive effect with additional chemoprevention by melatonin and metformin on cardiac lysosomes. It should be noted that the NAG enzyme activity was characterized by a similar synergistic effect on the hepatic and splenic tissues in the two models of carcinogenesis (Table 2).

Β-galactosidase (β-GD) was found to be able to catalyze two different types of reactions, namely hydrolysis and transgalactosylation and to be effective in the proliferation of breast cancer cells^43^. Therefore, we decided to analyze a lysosomal exoglycosidase involved in the catabolism of glycoconjugates in the different models of carcinogenesis induction with additional chemoprevention by MEL and MF using various animal tissues (Table 3). Elevated activity of another enzyme, i.e. beta-glucuronidase (β-GR), is associated with an increased risk of various cancers, particularly hormone-dependent breast, prostate, bladder, and colon cancers^44,45^. Its activity was determined in the next part of our investigation. The value of β-GR in the lysosomes of the three selected tissues is shown in Table 3.

**Table 3:** Effect of melatonin and metformin on β-glucuronidase and β-galactosidase activity (nM/h/mg protein) in the liver, heart and spleen in female Sprague-Dawley rats subjected to a high-fat diet in the mammary carcinogenesis model induced by *N*-methyl-*N*-nitrosourea (NMU) or dimethylbenzanthracene (DMBA). Metformin (MF) or melatonin (MEL) was administered alone and in combination (MF + MEL).

Parameters/ Groups	Liver	Heart	Spleen	Liver	Heart	Spleen
	β-GD, nmol h^−1^ mg^−1^ protein			β-GR, nmol h^−1^ mg^−1^ protein		
Control	313.06 ± 67.48	186.02 ± 17.58	184.73 ± 1.63	317.40 ± 45.70	62.24 ± 6.01	200.15 ± 2.14
NMU	575.14 ± 40.39^a^	180.57 ± 21.25	163.60 ± 21.14	613.04 ± 45.41^a^	102.80 ± 8.75^a^	177.28 ± 31.12
DMBA	478.66 ± 78.20^aa^	194.33 ± 22.14	170.07 ± 25.44	675.45 ± 112.01^aa^	132.81 ± 8.41^aa^	184.32 ± 21.17
MF + NMU	490.24 ± 50.60	214.76 ± 21.40	275.21 ± 24.18^b^	535.45 ± 57.89	77.41 ± 6.47^b^	259.09 ± 25.41^b^
MF + DMBA	465.22 ± 58.12	198.22 ± 15.41	247.51 ± 32.41^bb^	455.87 ± 71.20	84.25 ± 8.69^bb^	214.21 ± 33.25
MEL + NMU	443.52 ± 64.58^c^	209.52 ± 62.11	225.19 ± 36.01	575.84 ± 58.58	57.54 ± 8.42	239.11 ± 21.78
MEL + DMBA	585.02 ± 74.02^cc^	205.21 ± 17.01	337.04 ± 47.91^cc^	498.47 ± 62.52	51.25 ± 5.01^cc^	205.63 ± 19.09
MF + MEL + NMU	317.70 ± 44.96^d,e^	168.33 ± 15.42^d^	188.39 ± 17.96^d^	387.52 ± 60.21^d,e^	55.90 ± 3.09^d^	177.64 ± 17.05^d^
MF + MEL + DMBA	314.08 ± 18.12^ee^	162.58 ± 24.17	172.15 ± 17.91^ee^	390.54 ± 68.21	53.40 ± 5.58^dd^	162.24 ± 21.47

Note. Data are expressed as means ± S.D.

Significant differences *P* < 0.05 are designated as follows: a—NMU group vs. control group; aa—DMBA group vs. control group; b—MF + NMU vs. NMU group; bb—MF + DMBA vs. DMBA group; c—MEL + NMU vs. NMU group; cc—MEL + DMBA vs. DMBA group; d—MEL + MF + NMU vs. MF + NMU group; dd—MEL + MF + DMBA vs. MF + DMBA group; e—MEL + MF + NMU vs. MEL + NMU group; ee—MEL + MF + DMBA vs. MEL + DMBA group.

β-GD, β-galactosidase; β-GR, β-glucuronidase.

NMU, *N*-methyl-*N*-nitrosourea; DMBA, dimethylbenzanthracene; MF, metformin; MEL, melatonin.

In our studies, we observed tissue-specific dependence of the activity of β-GD in both models of NMU- and DMBA-induced carcinogenesis with the high-fat diet impact in animals (Table 3). Compared to the control, a statistically significant increase in the activity of the enzyme was noted only in the hepatic tissue. MEL was found to exert different effects on the activity of β-GD in the liver lysosomes: it reduced the β-GD activity level in the MEL + NMU model and increased the activity of the enzyme in the MEL + DMBA model, in comparison with the NMU and DMBA groups. In the case of the splenic tissue subjected to the MF chemoprevention in both models, the activity of β-GD was statistically significantly higher in comparison with the MF-untreated models of carcinogenesis induction in rats. A synergistic limiting effect of MEL and MF on the destructive processes of lysosomal β-GD enzyme activity was obtained in both models of carcinogenesis in the case of the three tissues (Table 3). A statistical increase in β-GR enzyme activity in the hepatic and cardiac tissues was noted in both models of NMU- and DMBA-induced chemical carcinogenesis with the high-fat diet impact. The resulting degradation of lysosomal function was minimized with the use of the MEL and MF chemoprevention and was tissue specific, as shown in Table 3. We obtained synergistic effects of MEL and MF, which resulted in positive correction of changes induced by lysosomal destruction processes, as shown by the β-GR activity in the hepatic, cardiac, and splenic tissues. These changes were statistically significant only in the case of the cardiac tissue in the two models of induced carcinogenesis.

### Oxidative stress biomarkers

**Figure 1:**
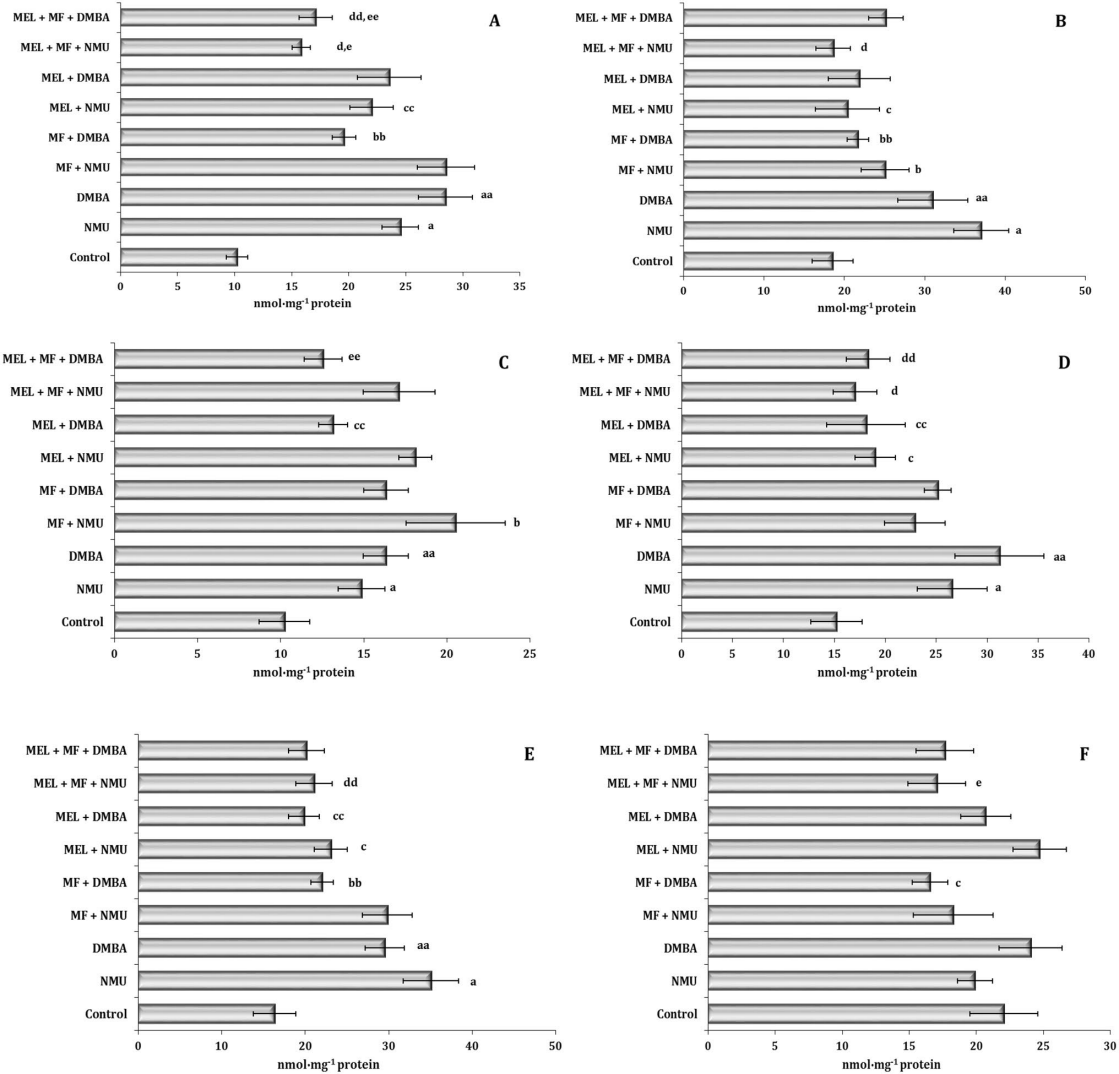
Content of conjugated dienes (A_233_, nmol mg^−1^ protein) in the liver (**A**), heart (**B**), spleen (**C**) and TBARS (nmol mg^−1^ protein) in the liver (**D**), heart (**E**), and spleen (**F**) of female Sprague–Dawley rats subjected to a high-fat diet in the mammary carcinogenesis model induced by *N*-methyl-*N*-nitrosourea (NMU) or dimethylbenzanthracene (DMBA). Metformin (MF) or melatonin (MEL) was administered alone and in combination (MF + MEL). Data are expressed as means ± S.D. Significant differences *P* < 0.05 are designated as follows: a—NMU group vs. control group; aa—DMBA group vs. control group; b—MF + NMU vs. NMU group; bb—MF + DMBA vs. DMBA group; c—MEL + NMU vs. NMU group; cc—MEL + DMBA vs. DMBA group; d—MEL + MF + NMU vs. MF + NMU group; dd—MEL + MF + DMBA vs. MF + DMBA group; e—MEL + MF + NMU vs. MEL + NMU group; ee—MEL + MF + DMBA vs. MEL + DMBA group. NMU, *N*-methyl-*N*-nitrosourea; DMBA, dimethylbenzanthracene; MF, metformin; MEL, melatonin.

The development and induction of the carcinogenesis process in the different rat models with the high-fat diet impact were also accompanied by initiation of free-radical oxidation processes, which we studied at the initial (estimated by the level of diene conjugates) and final (TBARS products) stages of this process. The data for the different tissues analyzed in this series of studies are presented in Fig. 1A–F. The level of initial lipid peroxidation processes (assessed by diene conjugation) showed a statistically significant increase in their intensity in the cardiac tissue in the NMU model and in the hepatic tissue in the DMBA model (Fig. 1A and B). The treatment with MEL and MF in the different experimental conditions had a pronounced protective chemopreventive effect depending on the tissue type. The increase in the content of the initial and end products of the lipid peroxidation process was predominantly pronounced in the hepatic and cardiac tissues and in the splenic tissue only in the initial stages of this process.

**Figure 2:**
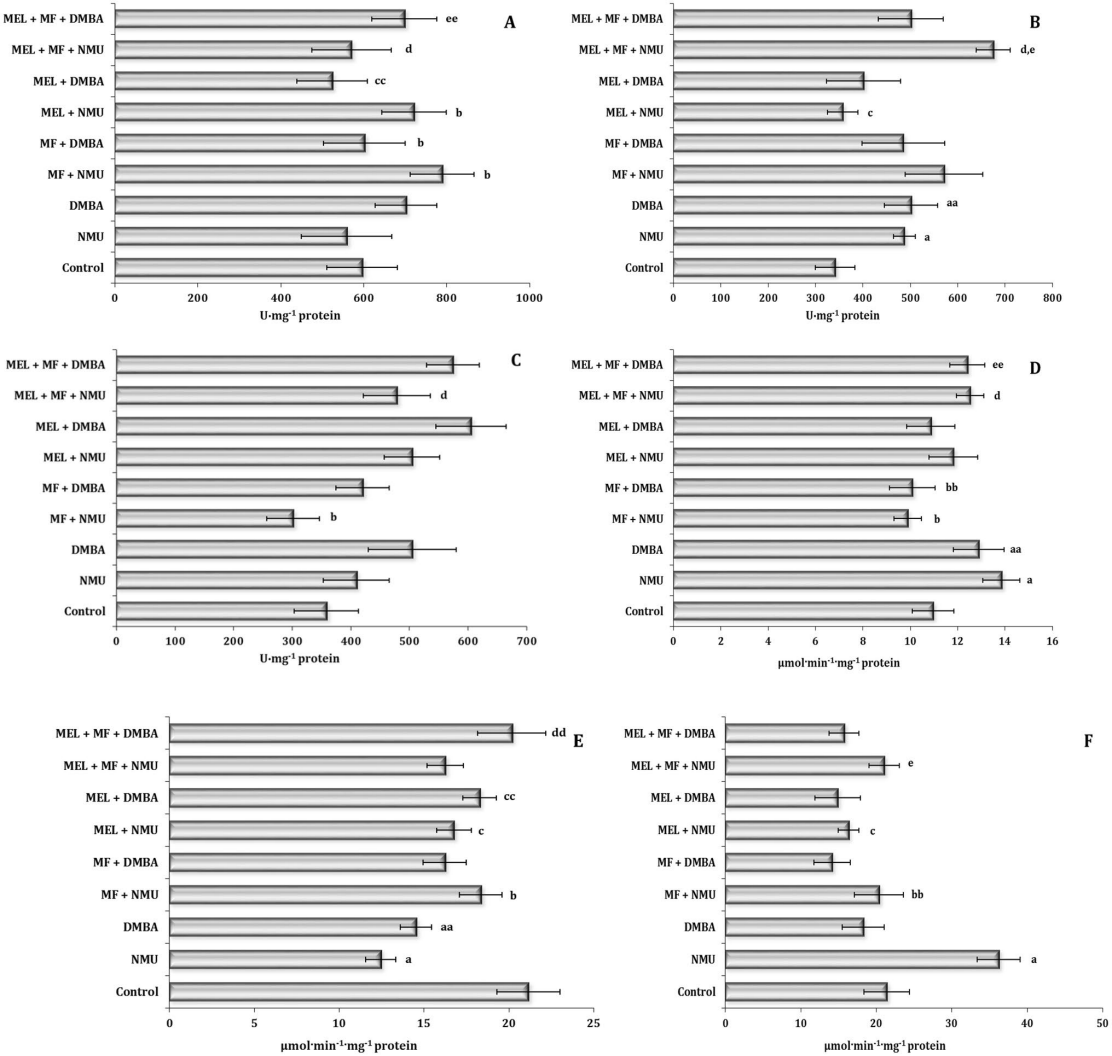
Superoxide dismutase activity (U mg^−1^ protein) in the liver (**A**), heart (**B**), spleen (**C**) and catalase activity (μmol min^−1^·mg^−1^ protein) in the liver (**D**), heart (**E**) and spleen (**F**) of female Sprague–Dawley rats subjected to a high-fat diet in the mammary carcinogenesis model induced by *N*-methyl-*N*-nitrosourea (NMU) or dimethylbenzanthracene (DMBA). Metformin (MF) or melatonin (MEL) was administrated alone and in combination (MF + MEL). Data are expressed as means ± S.D. Significant differences *P* < 0.05 are designated as follows: a—NMU group vs. control group; aa—DMBA group vs. control group; b—MF + NMU vs. NMU group; bb—MF + DMBA vs. DMBA group; c—MEL + NMU vs. NMU group; cc—MEL + DMBA vs. DMBA group; d—MEL + MF + NMU vs. MF + NMU group; dd—MEL + MF + DMBA vs. MF + DMBA group; e—MEL + MF + NMU vs. MEL + NMU group; ee—MEL + MF + DMBA vs. MEL + DMBA group. NMU, *N*-methyl-*N*-nitrosourea; DMBA, dimethylbenzanthracene; MF, metformin; MEL, melatonin.

**Figure 3:**
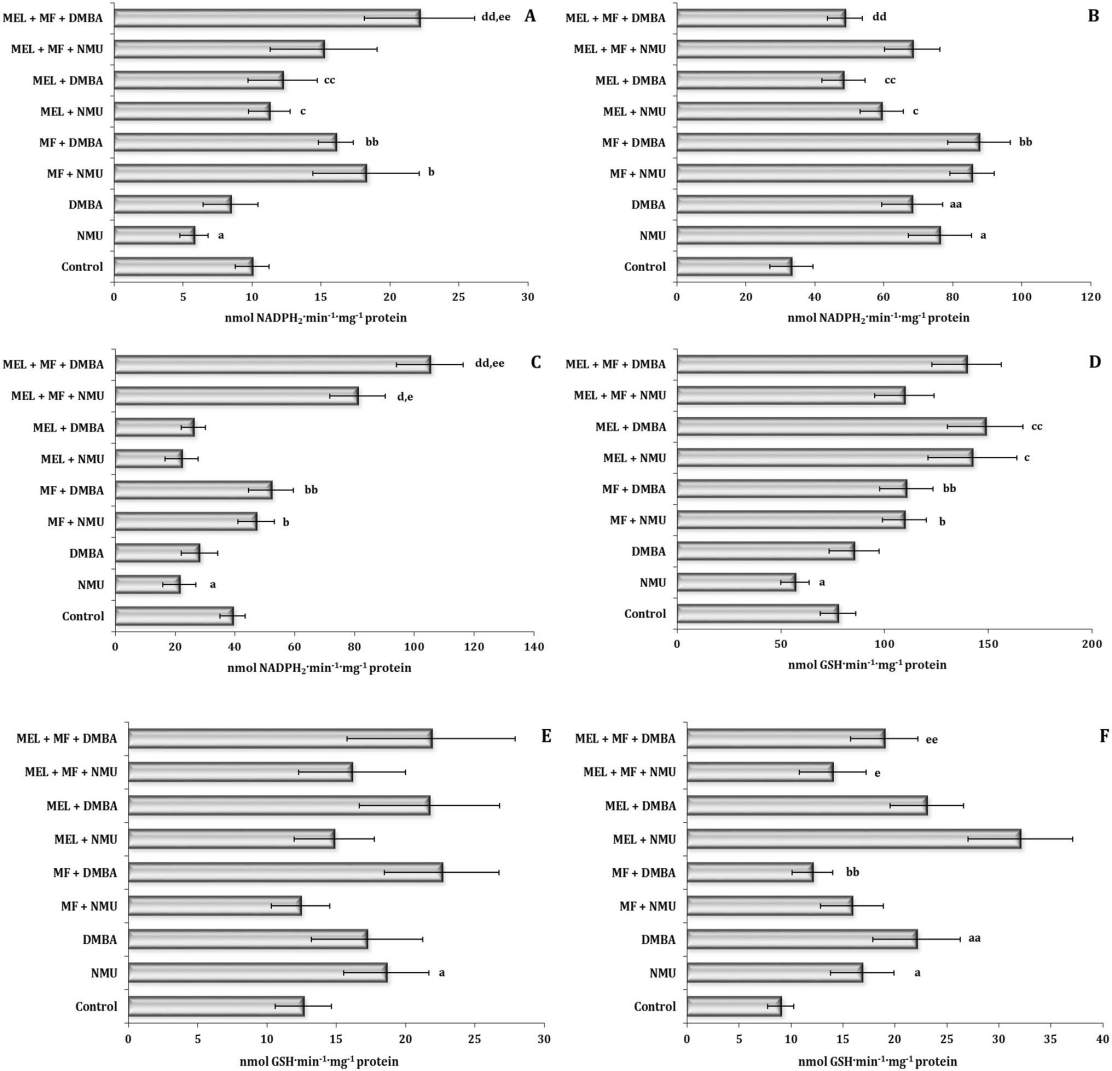
Glutathione reductase activity (nmol NADPH_2_·min^−1^·mg^−1^ protein) in the liver (**A**), heart (**B**) and spleen (**C**) and glutathione peroxidase activity (nmol GSH ·min^−1^·mg^−1^ protein) in the liver (**D**), heart (**E**) and spleen (**F**) of female Sprague–Dawley rats subjected to a high-fat diet in the mammary carcinogenesis model induced by *N*-methyl-*N*-nitrosourea (NMU) or dimethylbenzanthracene (DMBA). Metformin (MF) or melatonin (MEL) was administrated alone and in combination (MF + MEL). Data are expressed as means ± S.D. Significant differences *P* < 0.05 are designated as follows: a—NMU group vs. control group; aa—DMBA group vs. control group; b—MF + NMU vs. NMU group; bb—MF + DMBA vs. DMBA group; c—MEL + NMU vs. NMU group; cc—MEL + DMBA vs. DMBA group; d—MEL + MF + NMU vs. MF + NMU group; dd—MEL + MF + DMBA vs. MF + DMBA group; e—MEL + MF + NMU vs. MEL + NMU group; ee—MEL + MF + DMBA vs. MEL + DMBA group. NMU, *N*-methyl-*N*-nitrosourea; DMBA, dimethylbenzanthracene; MF, metformin; MEL, melatonin.

The induction of oxidative stress in the two different models of carcinogenesis with the high-fat diet impact showed high tissue specificity of this process associated with the activity of the main antioxidant enzymes of the first (superoxide dismutase, SOD) as shown in Fig. 2A–C and second level of cell protection (catalase, CAT) (Fig. 2D–F) and enzymes of glutathione metabolism (glutathione reductase, GR and glutathione peroxidase, GPx) (Fig. 3) depending on the type of the chemical exposure model. Our results showed that the initiation of oxidative stress in the cardiac tissue was accompanied by a statistically significant increase in SOD activity and a decrease in CAT activity in both models of carcinogenesis. In the case of the hepatic tissue, we observed a statistically significant increase in CAT activity with no change in SOD activity in the NMU and DMBA models. In the liver, only the NMU model exhibited a significant increase in CAT activity. The effects of MEL and MF applied alone or in combination in each animal model of carcinogenesis were specific and caused statistically significant synergistic interactions in each of the three studied tissues (Figs. 2A–F and 3A–F).

## Discussion

The relevance of our study is to find out the role of specific biomarkers of lysosome destruction and oxidative stress data in carcinogenesis models. The objective of the current work was to investigate and compare the synergic effect of peroral antidiabetic MF and pineal hormone MEL administered alone and in combination in two different rat models of mammary tumor proliferation in vivo on lysosomal destruction processes caused by oxidative stress in three types of tissues differing in the metabolism and proliferation value. To our knowledge, this is the first study to investigate the relationship between lysosome functioning in different types of rat mammary tumor and oxidative biomarkers. Previous studies showed a large variance in tumor parameters, depending on the size of the examined cancer models, severity of the disease, measurement period, and measurement methods^27,46^. The research team from Slovakia repeatedly reported preventive effects of MEL administered alone and in combination with other chemopreventives in female rat mammary carcinogenesis. The results indicate that the preventive-curative treatment (i.e. melatonin administration before and after carcinogenesis induction) is the most suitable approach, as proved by the increased latency and survival time, and a combination with other oncostatic substances is preferable^47,48^.

A series of studies have convincingly demonstrated the role of polycyclic aromatic hydrocarbons as potent carcinogens. Among these compounds, DMBA is well known for its capacity to induce mammary carcinomas in female Sprague–Dawley rats. Since ovariectomy was shown to suppress the susceptibility to DMBA^49^, it can be suggested that the inducible action of the carcinogen depends on ovarian hormones. The promotion of DMBA-induced adenocarcinoma is accompanied by a series of neuroendocrine disruptions of both hypothalamic–pituitary–gonadal and hypothalamic–pituitary–adrenal axes. Importantly, the secretion of melatonin during the 2-month latency period in these conditions precedes the occurrence of the first mammary tumor. The DMBA effects have shown possible relationships between neuroendocrine disruptions, which essentially consist in an increased pre-ovulatory secretion of such hormones like estradiol and prolactin, associated with a marked reduction of MEL synthesis^49^. This may be the reason why complementary therapy with MEL, which has a wide range of physiological effects^16^, is important for reduction of the incidence of malignancy and mortality in diagnosed patients who have taken MF for their diabetes mellitus. In addition, MEL is a strong antioxidant, as the indole part of its molecule interacts with free radicals and neutralizes them by activating antioxidant protection enzymes. Furthermore, it has hepatoprotective properties and exhibits an immunomodulatory effect^28^.

The present original experimental research is focused on comparative analysis of the use of different models of carcinogenesis induction undertaken mainly to provide new knowledge of the underlying foundations of the synergic effect of MF and MEL. It should be noted that the effects of each of the pharmacological agents under consideration have been reported in the literature^11,12,17^; however, we compared their effects using efficient and sensitive markers of cellular damage, i.e. the mechanisms of lysosomal destruction induced by oxidative stress. These types of comparisons are less common in the literature and are still noteworthy.

There are several novel findings in our study. Our results revealed an increase in the activity of the studied lysosomal enzymes (alanyl aminopeptidase, leucyl aminopeptidase, acid phosphatase, β-*N*-acetylglucosaminidase, β-galactosidase, and β-glucuronidase), whose expression was tissue-specific (liver, heart, and spleen) depending on the type of the chemical agent (NMU or DMBA). Such selectivity can be based on the selective induction of death, apoptosis, autophagy, or necroptosis in cancer cells^50^. Autophagy can promote both cell survival under stressors by recycling damaged organelles, misconfiguration of particle molecules, or cell death by over-activation of digestion of cellular components and degradation of key structures under enforced oxidative stress^51^. In extensive induced damage, the cell dies by autophagy^52^. Potential drugs can be partner molecules of already existing and widely used drugs, allowing their action to be enhanced synergistically. Recently, the role of biomarkers of specific lysosomal enzyme complexes has been reported in a number of papers. They described the role of AAP in solid and hematologic tumors^37,38^, LAP in the progression and metastasis of several cancers^39,40^, AcP as a link between chronic inflammation and breast cancer^41^, NAG in luminal breast epithelial cells^42^, and β-GD in the proliferation of breast cancer cells^43^.

Disturbances in tissue ultrastructure caused by chemical stressors during induction of the carcinogenesis process can be associated with increased oxidative stress^50^, as shown by us in the previous and this study, with a decrease in energy sources and metabolic reserves, damage to protein synthesis and energy apparatus (cristae and matrix disorganization in mitochondria or destruction), disruption of carbohydrate and lipid exchange, cell destruction and necrosis, spatial reorganization, changes in blood vessel structure and blood filling, and initiation of inflammation^18,20^.

The MANOVA tests used in our study confirmed the influence of the three main factors, i.e. the type of tissue, chemical impact, and type of chemopreventive agents, and the combinations of these factors on the lysosomal activity induced during the process of carcinogenesis. The alanyl aminopeptidase activity was dependent on the tissue type and the chemopreventive agents. The leucyl aminopeptidase value was influenced by the type of tissue and the carcinogenesis-inducing chemical agents. Acid phosphatase was correlated statistically significantly with all three factors: tissue type, carcinogenesis model, and chemopreventive agents. The MANOVA sum of the square test allowed us to draw the following conclusions on the role of each lysosomal parameter analyzed as an integral model. These dependencies are as follows: NAG > AcP > β-GD > β-GR > AAP > LAP.

Our results confirm the hypothesis that dysregulation of oxidative stress metabolism in breast cancer is associated with enhanced activity of lysosomal enzymes. Activation of lipid peroxidation induced by chemical stressors during carcinogenesis induction was observed at both the initial and final stages of the process, and the observed correlations between the oxidative stress parameters and lysosomal enzyme activity were statistically significant for the studied carcinogenesis models in most cases for the cardiac and hepatic tissues. In the NMU model of carcinogenesis, the following relationships were determined: AcP—TBARS (r = 0.85, *p* = 0.000) in the liver, NAG—DK (r = 0.76, *p* = 0.001) in the heart, and β-GD—TBARS (r = 0.81, *p* = 0.000) in the liver. In the DMBA model of carcinogenesis, the following associations were indicated: AcP—TBARS (r = 0.88, *p* = 0.000) and NAG—TBARS (r = 0.78, *p* = 0.000) in the liver, β-GR—DK (r = 0.69, p = 0.001) in the liver, and β-GR—TBARS (r = 0.82, *p* = 0.000) in the heart. The SS test allowed us to draw the following conclusions on the role of each analyzed parameter of oxidative stress biomarkers with antioxidant enzyme activity analyzed as an integral statistical model. These dependencies are as follows: TBARS > CAT > DK > SOD > GR > GPx.

We observed statistically significant activation of the studied lysosomal enzymes in our experiment in both stress models induced by the exposure to the chemical stressors. These changes were dependent on the tissue type, the form of the lysosomal enzymes (e.g. membrane-bound, as for AAP), and the type of carcinogenesis-inducing reagent. We have found the following statistically significant effects in the NMU experiment: AAP in the spleen, LAP in the liver, AcP in the liver and spleen, NAG in all three tissues, β-GD in the liver, and β-GR in the liver and heart. Similar effects were observed for enzyme activities in the DMBA variant: AAP in the spleen and heart, LAP in the liver, AcP in the liver and spleen, NAG in the liver and spleen, β-GD in the liver, and β-GR in the liver and heart. Some authors reported promising efficacy of glucuronide prodrugs in anti-cancer therapy related to their increased specificity and reduced systemic toxicity. The prodrugs can be used in prodrug monotherapy, which is based on elevated tumor β-glucuronidase activity^53^. It has been shown that β-GR activates low-toxic prodrugs into highly cytotoxic agents specifically in the tumor site. The specificity of the prodrugs can be further improved through a combined use with monoclonal antibodies against tumor-specific antigens, namely antibody-directed enzyme prodrug therapy. The potency of the prodrugs can be greatly enhanced by incorporation of combined chemo- and radio-therapy in anti-cancer strategy^53^.

Our data on the prevention of lysosomal dysfunction and limiting the development of oxidative stress depending on the tissue and reagent type will MF and MEL have provided new facts on the efficacy of peroral antidiabetics and possibilities to enhance their effects in combination with MEL, i.e. a well-known oncostatic substance, in in vivo mammary carcinogenesis models.

A recent study has demonstrated the oncostatic effect of MEL on chemically induced mammary carcinogenesis in rats. It was found that MEL administered at a dose of 2.5 mg/kg on the same day with DMBA significantly reduced the incidence of mammary adenocarcinoma from 79 to 20% in Sprague–Dawley rats^54^. More recently, it has been shown that MEL administered at a dose of 100 µg/day 30 days before and 90 days after exposure to DMBA significantly reduced the incidence of mammary adenocarcinomas and increased the latent period of tumor growth in Holtzman rats with a removed pineal gland^55^. MEL administered at a dose of 500 µg/day in combination with 13 cis-retinoic acid was found to increase apoptosis and cause regression of mammary tumors induced by nitrosomethylurea (NMU) in Sprague–Dawley rats^56^. In another study, MEL was administered at a dose of 10 mg/kg/day to female Sprague–Dawley rats 15 days before DMBA carcinogen exposure or later. A reduction in the incidence of mammary adenocarcinomas compared to controls was observed in rats that received MEL for preventive purposes before the carcinogen exposure and in animals that received MEL for therapeutic purposes^57^. A more recent study has also demonstrated the effect of 20 mg/l MEL administered with drinking water on DMBA-induced mammary carcinogenesis in rats receiving a high-fat diet. The duration of MEL administration was 20 days before and 14 weeks after the carcinogen exposure. The incidence of mammary tumors was lower in the group of animals receiving MEL than in the control group. Immunohistochemical examination of tumors that developed in animals receiving MEL showed increased expression of apoptosis markers (caspase-3, BAX)^58^.

A number of studies have shown that both MEL and MF have antitumor activity in vitro against different tumor types, in particular, breast cancer cell lines^59^. They can inhibit spontaneous and chemically induced carcinogenesis of breast cancer in vivo^55^ and tumor growth in models of transplanted tumors and breast tumor xenografts^60^. Epidemiological studies show a reduction in the incidence of malignant tumors and mortality in patients who have taken MF in association with diabetes mellitus. In particular, a 17–20% reduction in the risk of breast cancer was observed in diabetic patients who received MF, compared to those who did not receive the drug^61^. Several studies have reported benefits of the use of MF for overall and recurrence-free survival after radical treatment of breast cancer patients^62^.

## Conclusions

The current study was focused on a comparative analysis of the synergistic effects of MEL and MF administration in two different cancer models (NMU and DMBA) on lysosomal enzyme status in relation to the induction of oxidative stress in different types of tissues differing in their sensitivity to oncostatic agents due to the different levels of metabolic processes, hematopoiesis intensity, and morphological characteristics of cells. We analyzed the effect of MF administered alone and in combination with MEL in chemically induced mammary carcinogenesis in female Sprague–Dawley rats fed a high-fat diet. Our results revealed an increase in the activity of the studied lysosomal enzymes (alanyl aminopeptidase AAP, leucyl aminopeptidase LAP, acid phosphatase AcP, β-*N*-acetylglucosaminidase NAG, β-galactosidase β-GD, and β-glucuronidase β-GR), whose expression was tissue-specific (liver, heart and spleen). Depending on the type of chemical agent (NMU or DMBA), lysosomes can simultaneously induce various types of cancer cell death, i.e. apoptosis, autophagy, or necroptosis. Our results confirm the hypothesis that dysregulation of oxidative stress metabolism in breast cancer is associated with enhanced activity of lysosomal enzymes, as corroborated by the correlative analysis. The MANOVA tests used in our study confirmed the influence of the three main factors, i.e. the type of tissue, chemical impact, and type of chemopreventive agents, and the combinations of these factors on the lysosomal activity induced during the process of carcinogenesis. The combined application of MEL and MF in the two models of carcinogenesis with the high-fat diet impact showed a significant synergistic effect on AAP, compared with the effects of one substance administered alone (either MEL or MF) in the breast cancer model experiments. We observed a marked (threefold) increase in LAP enzyme activity in the liver tissue without impairment of this enzyme in the heart and spleen lysosomes. The activation of membrane-dependent processes of lysosomal LAP functioning in the liver tissue was reduced statistically significantly in the series of experiments using MF and MEL in the NMU model and MEL in the DMBA model. We have identified tissue-dependent effects on the AcP functioning in lysosomes in the model of NMU- and DMBA-induced carcinogenesis with the high-fat diet impact. In the liver and spleen tissue, we showed twofold and higher levels of statistically significant increases in AcP activity depending on the type of the cancer model. A synergistic limiting effect of MEL and MF on the destructive processes of lysosomal β-GD enzyme activity was obtained in both models of carcinogenesis in the case of the three tissues. The SS test allowed us to draw the following conclusions on the role of each lysosomal parameter analyzed as an integral model: NAG > AcP > β-GD > β-GR > AAP > LAP.

## Supplementary Information

Supplementary Information.

## Data Availability

The data presented in this study are available on request from the corresponding author.

## References

[1] Harbeck N, Gnant M (2017). Breast cancer. Lancet.

[2] Ferlay J, Steliarova-Foucher E, Lortet-Tieulent J, Rosso S, Coebergh JWW, Comber H, Forman D, Bray F (2013). Cancer incidence and mortality patterns in Europe: Estimates for 40 countries in 2012. Europ. J. Cancer..

[3] Radecka B, Litwiniuk M (2016). Breast cancer in young women. Ginekol. Pol..

[4] Kurhaluk N, Bojková B, Kajo K, Macháleková K, Kisková T (2021). Addition of palm olein to lard-supplemented diet indicates myocardial dysfunction and augments oxidative stress by authophagy-lysosome pathway in rats. J. Anim. Physiol. Anim. Nutr. (Berl.).

[5] Nagini S (2017). Breast cancer: Current molecular therapeutic targets and new players. Anticancer Agents Med. Chem..

[6] Faria J, Negalha G, Azevedo A, Martel F (2019). Metformin and breast cancer: Molecular targets. J. Mammary Gland Biol. Neoplasia..

[7] De A, Kuppusamy G (2020). Metformin in breast cancer: Preclinical and clinical evidence. Curr Probl Cancer..

[8] Coyle C, Cafferty FH, Vale C, Langley RE (2016). Metformin as an adjuvant treatment for cancer: A systematic review and meta-analysis. Ann Oncol..

[9] Reiter RJ, Mayo JC, Tan DX, Sainz RM, Alatorre-Jimenez M, Qin L (2016). Melatonin as an antioxidant: Under promises but over delivers. J. Pineal Res..

[10] Cipolla-Neto J, Amaral FGD (2018). Melatonin as a hormone: New physiological and clinical insights. Endocr. Rev..

[11] Kurhaluk N, Bojkova B, Radkowski M, Zaitseva OV, Kyriienko S, Demkow U, Winklewski PJ (2018). Melatonin and metformin diminish oxidative stress in heart tissue in a rat model of high fat diet and mammary carcinogenesis. Adv. Exp. Med. Biol..

[12] Kurhaluk N, Szarmach A, Zaitseva OV, Sliuta A, Kyriienko S, Winklewski PJ (2018). Effects of melatonin on low-dose lipopolysaccharide-induced oxidative stress in mouse liver, muscle, and kidney. Can. J. Physiol. Pharmacol..

[13] Meng X, Li Y, Li S, Zhou Y, Gan RY, Xu DP, Li HB (2017). Dietary sources and bioactivities of melatonin. Nutrients.

[14] Bhattacharya S, Patel KK, Dehari D, Agrawal AK, Singh S (2019). Melatonin and its ubiquitous anticancer effects. Mol. Cell Biochem..

[15] Samanta S (2020). Melatonin: An endogenous miraculous indolamine, fights against cancer progression. J. Cancer Res. Clin. Oncol..

[16] Kurhaluk N, Tkachenko H (2020). Melatonin and alcohol-related disorders. Chronobiol. Int..

[17] Kurhaluk N, Bojková B, Winklewski PJ (2018). Liver antioxidant and aerobic status improves after metformin and melatonin administration in a rat model of high-fat diet and mammary carcinogenesis. Can. J. Physiol. Pharmacol..

[18] Gurer-Orhan H, Ince E, Konyar D, Saso L, Suzen S (2018). The role of oxidative stress modulators in breast cancer. Curr. Med. Chem..

[19] Klaunig JE (2018). Oxidative stress and cancer. Curr. Pharm. Des..

[20] Reuter S, Gupta SC, Chaturvedi MM, Aggarwal BB (2010). Oxidative stress, inflammation, and cancer: how are they linked?. Free Radic. Biol. Med..

[21] Kurhaluk N, Zaitseva OV, Sliuta A, Kyriienko S, Winklewski PJ (2018). Melatonin diminishes oxidative stress in plasma, retains erythrocyte resistance and restores white blood cell count after low dose lipopolysaccharide exposure in mice. Gen. Physiol. Biophys..

[22] Zhitomirsky B, Assaraf YG (2017). Lysosomal accumulation of anticancer drugs triggers lysosomal exocytosis. Oncotarget.

[23] Lee J, Giordano S, Zhang J (2012). Autophagy, mitochondria and oxidative stress: cross-talk and redox signalling. Biochem. J..

[24] Tang T, Yang ZY, Wang D, Yang XY, Wang J, Li L, Wen Q, Gao L, Bian XW, Yu SC (2020). The role of lysosomes in cancer development and progression. Cell Biosci..

[25] Huang T, Song X, Yang Y, Wan X, Alvarez AA, Sastry N, Feng H, Hu B, Cheng SY (2018). Autophagy and hallmarks of cancer. Crit. Rev. Oncog..

[26] Zhang J, Wang G, Zhou Y, Chen Y, Ouyang L, Liu B (2018). Mechanisms of autophagy and relevant small-molecule compounds for targeted cancer therapy. Cell Mol. Life Sci..

[27] Bojková B, Kajo K, Kubatka P, Solár P, Péč M, Adamkov M (2019). Metformin and melatonin improve histopathological outcome of NMU-induced mammary tumors in rats. Pathol. Res. Pract..

[28] Kurhaluk N (2021). Alcohol and melatonin. Chronobiol. Int..

[29] DeMartino GN, Goldberg AL (1978). Thyroid hormones control lysosomal enzyme activities in liver and skeletal muscle. Proc. Natl. Acad. Sci. USA.

[30] Kamyshnikov VS (2004). Reference book on clinic and biochemical researches and laboratory diagnostics.

[31] Kostiuk VA, Potapovich AI, Kovaleva ZhV. Prostoĭ i chuvstvitel'nyĭ metod opredeleniia aktivnosti superoksid- dismutazy, osnovannyĭ na reaktsii okisleniia kvertsetina [A simple and sensitive method of determination of superoxide dismutase activity based on the reaction of quercetin oxidation]. *Vopr. Med. Khim*. 1990; **36**(2): 88–91. Russian. 2363268

[32] Koroliuk MA, Ivanova LI, Maĭorova IG, Tokarev VE. Metod opredeleniia aktivnosti katalazy [A method of determining catalase activity]. *Lab Delo*. 1988; (1): 16–19. Russian. 2451064

[33] Glatzle D, Vuilleumier JP, Weber F, Decker K (1974). Glutathione reductase test with whole blood, a convenient procedure for the assessment of the riboflavin status in humans. Experientia.

[34] Moin VM. Prostoĭ i spetsificheskiĭ metod opredeleniia aktivnosti glutationperoksidazy v éritrotsitakh [A simple and specific method for determining glutathione peroxidase activity in erythrocytes]. *Lab. Delo*. 1986; (12): 724–727. Russian. 2434712

[35] McDonald JK, Barrett AJ. Exopeptidases. In: *Mamamlian Proteases: A glossary and Bibliography.* Academic Press, 1986; 114–144.

[36] Barrett AJ, Heath MF, Dingle JT (1977). Lysosomal enzymes. Lysosomes, a Laboratory Handbook.

[37] Bauvois B, Dauzonne D (2006). Aminopeptidase-N/CD13 (EC 3.4.11.2) inhibitors: Chemistry, biological evaluations, and therapeutic prospects. Med. Res. Rev..

[38] Wickström M, Larsson R, Nygren P, Gullbo J (2011). Aminopeptidase N (CD13) as a target for cancer chemotherapy. Cancer Sci..

[39] Yang H, Dai G, Wang S, Zhao Y, Wang X, Zhao X, Zhang H, Wei L, Zhang L, Guo S, Song W, Guo L, Fang C (2020). Inhibition of the proliferation, migration, and invasion of human breast cancer cells by leucine aminopeptidase 3 inhibitors derived from natural marine products. Anticancer Drugs..

[40] Fang C, Zhang J, Yang H, Peng L, Wang K, Wang Y, Zhao X, Liu H, Dou C, Shi L, Zhao C, Liang S, Li D, Wang X (2019). Leucine aminopeptidase 3 promotes migration and invasion of breast cancer cells through upregulation of fascin and matrix metalloproteinases-2/9 expression. J. Cell Biochem..

[41] Chen YG, Janckila A, Chao TY, Yeh RH, Gao HW, Lee SH, Yu JC, Liao GS, Dai MS (2015). Association of tartrate-resistant acid phosphatase-expressed macrophages and metastatic breast cancer progression. Medicine (Baltimore).

[42] Ramessur KT, Greenwell P, Nash R, Dwek MV (2010). Breast cancer invasion is mediated by beta-*N*-acetylglucosaminidase (beta-NAG) and associated with a dysregulation in the secretory pathway of cancer cells. Br. J. Biomed. Sci..

[43] Vidya B, Palaniswamy M, Angayarkanni J, Ayub Nawaz K, Thandeeswaran M, Krishna Chaithanya K, Tekluu B, Muthusamy K, Gopalakrishnan VK (2020). Purification and characterization of β-galactosidase from newly isolated *Aspergillus terreus* (KUBCF1306) and evaluating its efficacy on breast cancer cell line (MCF-7). Bioorg. Chem..

[44] Paigen K, Peterson J, Paigen B (1984). Role of urinary beta-glucuronidase in human bladder cancer. Cancer Res..

[45] Heerdt AS, Young CW, Borgen PI (1995). Calcium glucarate as a chemopreventive agent in breast cancer. Isr. J. Med. Sci..

[46] Bojková B, Kajo K, Kisková T, Kubatka P, Žúbor P, Solár P, Péč M, Adamkov M (2018). Metformin and melatonin inhibit DMBA-induced mammary tumorigenesis in rats fed a high-fat diet. Anticancer Drugs..

[47] Bojková B, Garajová M, Péč M, Kubatka P, Kajo K, Mokáň M, Kassayová M, Orendáš P, Kisková T, Ahlersová E, Ahlers I (2011). Metabolic effects of pioglitazone in chemically-induced mammary carcinogenesis in rats. Pathol. Oncol. Res..

[48] Kubatka P, Bojková B, Mciková-Kalická K, Mníchová-Chamilová M, Adámeková E, Ahlers I, Ahlersová E, Cermáková M (2001). Effects of tamoxifen and melatonin on mammary gland cancer induced by *N*-methyl-*N*-nitrosourea and by 7,12-dimethylbenz(a)anthracene, respectively, in female Sprague-Dawley rats. Folia Biol. (Praha).

[49] Kerdelhué B, Forest C, Coumoul X (2016). Dimethyl-Benz(a)anthracene: A mammary carcinogen and a neuroendocrine disruptor. Biochim. Open..

[50] Lee J, Giordano S, Zhang J (2012). Autophagy, mitochondria and oxidative stress: Cross-talk and redox signalling. Biochem. J..

[51] Huang T, Song X, Yang Y, Wan X, Alvarez AA, Sastry N, Feng H, Hu B, Cheng SY (2018). Autophagy and hallmarks of cancer. Crit. Rev. Oncog..

[52] Zhang J, Wang G, Zhou Y, Chen Y, Ouyang L, Liu B (2018). Mechanisms of autophagy and relevant small-molecule compounds for targeted cancer therapy. Cell Mol. Life Sci..

[53] Chen X, Wu B, Wang PG (2003). Glucuronides in anti-cancer therapy. Curr. Med. Chem. Anticancer Agents..

[54] Tamarkin L, Cohen M, Roselle D, Reichert C, Lippman M, Chabner B (1981). Melatonin inhibition and pinealectomy enhancement of 7,12-dimethylbenz(a)anthracene-induced mammary tumors in the rat. Cancer Res..

[55] Kothari LS (1987). Influence of chronic melatonin on 9,10-dimethyl-1,2-benzanthracene-induced mammary tumors in female Holtzman rats exposed to continuous light. Oncology.

[56] Melancon K, Cheng Q, Kiefer TL, Dai J, Lai L, Dong C, Yuan L, Collins A, Thiyagarajah A, Long S, Hill SM (2005). Regression of NMU-induced mammary tumors with the combination of melatonin and 9-cis-retinoic acid. Cancer Lett..

[57] Lenoir V, de Jonage-Canonico MB, Perrin MH, Martin A, Scholler R, Kerdelhué B (2005). Preventive and curative effect of melatonin on mammary carcinogenesis induced by dimethylbenz[a]anthracene in the female Sprague-Dawley rat. Breast Cancer Res..

[58] Chuffa LG, Alves MS, Martinez M, Camargo IC, Pinheiro PF, Domeniconi RF, Júnior LA, Martinez FE (2016). Apoptosis is triggered by melatonin in an in vivo model of ovarian carcinoma. Endocr. Relat. Cancer..

[59] Anisimov VN (2015). Metformin for cancer and aging prevention: Is it a time to make the long story short?. Oncotarget.

[60] Jardim-Perassi BV, Arbab AS, Ferreira LC, Borin TF, Varma NR, Iskander AS, Shankar A, Ali MM, de Campos Zuccari DA (2014). Effect of melatonin on tumor growth and angiogenesis in xenograft model of breast cancer. PLoS ONE.

[61] Bosco JL, Antonsen S, Sørensen HT, Pedersen L, Lash TL (2011). Metformin and incident breast cancer among diabetic women: A population-based case-control study in Denmark. Cancer Epidemiol. Biomark. Prev..

[62] Sonnenblick A, Agbor-Tarh D, Bradbury I, Di Cosimo S, Azim HA, Fumagalli D, Sarp S, Wolff AC, Andersson M, Kroep J, Cufer T, Simon SD, Salman P, Toi M, Harris L, Gralow J, Keane M, Moreno-Aspitia A, Piccart-Gebhart M, de Azambuja E (2017). Impact of diabetes, insulin, and metformin use on the outcome of patients with human epidermal growth factor receptor 2-positive primary breast cancer: Analysis from the ALTTO Phase III Randomized Trial. J. Clin. Oncol..

