# Supplementary material for: Effects of melatonin and metformin in preventing lysosome-induced autophagy and oxidative stress in rat models of carcinogenesis and the impact of high-fat diet
Source: Sci Rep. 2022 Mar 23;12:4998. doi: 10.1038/s41598-022-08778-w (PMC8943031; doi:10.1038/s41598-022-08778-w)
Supplement: Supplementary file 1 — Supplementary Information. [file 41598_2022_8778_MOESM1_ESM.docx]

Table S1. Fatty acid composition of lard and palm oil diets (content of g/100 g)

| **Fatty acid** | **Lard** | **Palm oil** |
| --- | --- | --- |
| **Energy value**  Water  Cholesterol  **Saturated fatty acids**  C 10:0  C 12:0  C 14:0  C 15:0  C 16:0  C 17:0  C18:0  C 20:0  **Total**  **Monounsaturated fatty acids**  C 16:1  C 17:1  C 18:1  C 20:1  **Total**  **Polyunsaturated fatty acids**  C 18:2  C 18:3  20:4  **Total**  **Others:**  Minerals and vitamins:  Na  K  P  Fe  Zn  vitamin E  thiamine  B_6_ | 3682 kJ, 880 kcal  0.5 g  0.95.0  -  0.04  0.04  1.2  0.05  26.21  0.27  18.58  0.14  46.54  1.87  0.15  40.0  0.46  42.48  6.02  0.29  0.07  6.52  1 mg  1 mg  1 mg  0.1 mg  0.04 mg  1.2 mg  0.019 mg  0.18 mg | 3700 kJ, 884 kcal  fat 100 %  -  -  -  -  0.76  -  49.16  -  3.78  53.70  -  -  -  35.46  -  35.47  6.27 g  -  -  6.27  -  -  -  -  -  12 mg  -  - |

Data obtained from [Kunachowicz et al., 2005].
